# Risk factors and conservative therapy outcomes of anastomotic leakage after gastrectomy: Experience of 3,926 patients from a single gastric surgical unit

**DOI:** 10.3389/fonc.2023.1163463

**Published:** 2023-03-15

**Authors:** Zhongyuan He, Hongda Liu, Ling Zhou, Qingya Li, Linjun Wang, Diancai Zhang, Hao Xu, Zekuan Xu

**Affiliations:** ^1^ Department of General Surgery, The First Affiliated Hospital of Nanjing Medical University, Nanjing, China; ^2^ Department of Anesthesiology and Perioperative Medicine, The First Affiliated Hospital of Nanjing Medical University, Nanjing, China

**Keywords:** gastric cancer, anastomotic leakage, gastrectomy, complication, laparoscopic approach

## Abstract

**Background:**

Anastomotic leakage (AL) after gastrectomy is one of the severest postoperative complications and is related to increasing mortality. In addition, no consensus guidelines about strategies of AL treatment have been established. This large cohort study aimed to inspect the risk factors and efficacy of the conservative treatment for AL in patients with gastric cancer.

**Methods:**

We reviewed the clinicopathological data of 3,926 gastric cancer patients undergoing gastrectomy between 2014 and 2021. Results contained the rate, risk factors, and conservative therapy outcomes of AL.

**Results:**

In total, 80 patients (2.03%, 80/3,926) were diagnosed with AL, and esophagojejunostomy was the most frequent AL site (73.8%, 59/80). Among them, one patient (2.5%, 1/80) died. Multivariate analysis indicated that low albumin concentration (*P* = 0.001), presence of diabetes (*P* = 0.025), laparoscopic method (*P* < 0.001), total gastrectomy (*P* = 0.003), and proximal gastrectomy (*P* = 0.002) were predicting factors for AL. The closure rate for the conservative treatment of AL in the first month after AL diagnosis was 83.54% (66/79), and the median time from leakage diagnosis to the closure of leakage was 17 days (interquartile range 11–26 days). Low level of plasma albumin (*P* = 0.004) was associated with late leakage closures. In terms of 5-year overall survival, no significant difference was observed between patients with and without AL.

**Conclusion:**

The incidence of AL after gastrectomy is associated with low albumin concentration, diabetes, the laparoscopic method, and extent of resection. The conservative treatment is relatively safe and effective for the AL management in patients after gastric cancer surgery.

## 1 Introduction

Gastric cancer remains one of the most common cancers worldwide (1), but the mortality shows a continuously decreasing trend on account of the developments in surgical technique and perioperative management (2, 3). At present, radical gastrectomy is still the only probably curative therapy for resectable gastric cancer (4). Nevertheless, such surgical treatment includes the standard lymph node dissection and various reconstruction methods, and this high complexity of surgical procedure leads to a high risk of death and postoperative complications (5, 6). Anastomotic leakage (AL) is a destructive and potentially life-threatening postoperative complication, which is relevant to the increasing cost for treatment, the prolongation of hospitalization, and postoperative mortality (7–9). Moreover, several studies indicated that AL might shorten the long-term survival (10, 11).

The incidence of AL has been reported to be 1%~6% in gastric cancer patients after gastrectomy (12–14). Exploration of the risk factors for AL is vital to the prevention and therapy. Numerous studies had indicated that tumor location, sex, some comorbidities, neoadjuvant therapy, operation methods, surgeon’s experience, and combined resection might induce the occurrence of AL (15, 16). However, there was still no consensus on which of them are the decisive factors, resulting in the lack of pre- and postoperative optimization.

At present, methods of AL treatment were classified as surgery, endoscopic treatment, and conservative strategies (17). However, several studies suggested that reoperation, which was previously the first choice for AL management, increased the incidences of morbidity and mortality (11, 18). Along with the development of endoscopic techniques, such as self-expandable metallic stents (SEMS), endoscopic vacuum (EVAC) method, and stent-over-sponge (SOS) treatment, endoscopic treatment has brought about widespread attention in the past 20 years (19–21). Nevertheless, the superiority of endoscopic strategies, compared with conservative treatment, remains unclear. As such, this large cohort study was conducted to explore the risk factors and efficacy of conservative treatment for AL in patients with gastric cancer.

## 2 Materials and methods

### 2.1 Patients and study variables

Between January 2014 and December 2021, 3,926 patients with gastric cancer underwent gastrectomy at the First Affiliated Hospital of Nanjing Medical University, China. Electronic medical information of these patients was stored in a prospectively maintained database, and we collected all relevant information, containing baseline characteristics, clinical findings, intraoperative details, pathological data, and information about AL management. In this study, potential risk factors increasing the opportunities for AL were included as the following: age, sex, body mass index (BMI), American Society of Anesthesiologists (ASA) score, the concentrations of plasma hemoglobin, albumin, and cholesterol, the counts of neutrophil and lymphocyte, comorbidity, preoperative treatment, habits of smoking and drinking, history of previous laparotomy and neoadjuvant chemotherapy, tumor location, TNM stage, mode of surgery, radicality of surgery, type of resection and reconstruction, and duration of operation. The comparison between patients with and without AL based on above-mentioned electronic medical information. This study was approved by the Institutional Review of the First Affiliated Hospital of Nanjing Medical University.

### 2.2 Surgical procedures for gastrectomy

Over the course of the present study, the same surgical team performed total, distal, or proximal gastrectomy for these gastric cancer patients *via* open or laparoscopic approaches. Lymphadenectomy was performed with the D1+ or D2 extent according to the Japanese gastric cancer treatment (22). The methods of digestive tract reconstruction were used as the following: Billroth I, Billroth II, Roux-en-Y, uncut Roux-en-Y anastomosis for distal gastrectomy, Roux-en-Y esophagojejunostomy for total gastrectomy, and double-tract reconstruction or esophagogastrostomy for proximal gastrectomy. All types of anastomoses were performed using circular or linear staplers and were reinforced or not by manual suture.

### 2.3 Diagnosis of anastomotic leakage

Methods of AL diagnosis contain the assessment of clinical presentation, biochemical analysis, and diagnostic modalities, such as computed tomography (CT) scan, endoscopy, or fluoroscopy with radiocontrast. When patients had the clinical signs, including peritonitis, abnormal drainage from the abdominal drain catheter and fever, or a continuously high level of blood inflammatory biomarkers including blood cell counts, C-reactive protein (CRP), and procalcitonin, they were suspected of suffering from AL. Then, CT scan, taking methylene blue orally, or fluoroscopy was utilized to further confirm the presence of AL.

### 2.4 Treatment of anastomotic leakage

Most of patients initially underwent conservative treatment including fasting, continuous gastrointestinal decompression, enteral and parenteral nutrition support, and administration of broad-spectrum antibiotics. In case of abdominal abscess or hydrothorax, CT-guided percutaneous catheter drainage was performed to promote recovery of patients. When patients presented rapid clinical deterioration such as septic shock and hemorrhage, emergency operation was immediately considered. If the conservative treatment failed after 1 month or more, these patients might receive the endoscopic intervention depending on the decisions of the multidiscipline team.

### 2.5 Follow-up of the conservative treatment results

Biochemical analysis was performed to monitor the level of blood inflammatory biomarkers, including white blood cell counts, procalcitonin, and C-reactive protein every 3 days after the diagnosis of AL. Furthermore, CT scans with intravenous contrast were used to observe the absorption of intraperitoneal abscess and hydrothorax every 7 days or longer according to the clinical presentation of patients with AL. When the clinical signs such as fever or peritonitis disappeared, the level of blood inflammatory biomarkers became normal, the abdominal abscess and hydrothorax shrunk or even disappeared, and there was no abnormal drainage; the closure of AL was considered, and patients were discharged about a week later.

### 2.6 Statistical analysis

The Kolmogorov–Smirnov test was utilized to affirm the normal distribution of all variables. Non-parametric tests evaluated the non-normally distributed variables. Student’s *t*-test was used to analyze normally distributed variables. The interquartile range (IQR) or median with range presented the non-normally distributed variables, and the mean with standard deviation presented the continuous variables. Fisher’s exact test or chi-squared test was used to evaluate categorical variables. The multivariable Cox proportional hazard regression model further detected variables with significance in univariable analysis. The propensity score matching (PSM) method was utilized to balance the baseline features of two groups of patients with or without AL. Survival differences between patients with or without AL were investigated using the log-rank test, and the Kaplan–Meier method was used to analyze the survival data. Statistical significance was considered if *P* < 0.05. SPSS ver. 19.0 was used to perform all analyses.

## 3 Results

### 3.1 Incidence of anastomotic leakage

Overall, 3,926 gastric cancer patients undergoing gastrectomy between 2014 and 2021 were reviewed, and **Table 1** demonstrates the clinicopathological and operative features of these patients. A total of 80 patients (2.03%, 80/3,926) were eventually diagnosed with AL. Among them, one patient (1.25%, 1/80) died from severe septic shock 4 months after the surgery. Details of AL sites are presented in **Table 2**, and esophagojejunostomy (73.8%, 59/80) was remarkably more common than other AL sites.

**Table 1:** Clinicopathological and operative characteristics of the 3,926 patients who underwent gastrectomy.

Variable	
Age, years, number (%)	
<60	1,394 (35.5)
≥60	2,532 (64.5)
Sex, number (%)	
Male	2,845 (72.5)
Female	1,081 (27.5)
Body mass index, kg/m^2^, number (%)	
<25	2,789 (71.0)
≥25 and <30	1,031 (26.3)
≥30	106 (2.7)
ASA score, number (%)	
<3	3,335 (84.9)
≥3	591 (15.1)
Hemoglobin concentration, g/L, median (IQR)	130 (114-143)
Albumin concentration, g/L, median (IQR)	38.9 (36.2-41.7)
Total cholesterol, mmol/L, median (IQR)	4.46 (3.81-5.10)
Lymphocyte count, ×10^9^/L, median (IQR)	1.50 (1.20-1.84)
Neutrophil count, ×10^9^/L, median (IQR)	3.39 (2.70-4.34)
Diabetes, number (%)	
Absent	3,543 (90.2)
Present	383 (9.8)
Smoking habits, number (%)	
Non-smoker	2,827 (72.0)
Smoker	1,099 (28.0)
Drinking habits, number (%)	
Non-drinker	3,257 (83.0)
Drinker	669 (17.0)
Previously laparotomy, number (%)	
Absent	3,408 (86.8)
Present	518 (13.2)
Neoadjuvant chemotherapy, number (%)	
Absent	3,742 (95.3)
Present	184 (4.7)
Tumor location, number (%)	
Upper third stomach	1,146 (29.2)
Middle third stomach	1,127 (28.7)
Lower third stomach	1,456 (37.1)
others	197 (5.0)
T stage, number (%)	
T1	1,309 (33.3)
T2	515 (13.1)
T3	1,252 (31.9)
T4	850 (21.7)
N stage, number (%)	
N0	1,859 (47.4)
N1	552 (14.1)
N2	557 (14.2)
N3	958 (24.4)
Metastasis, number (%)	
M0	3,875 (98.7)
M1	51 (1.3)
Stage, number (%)	
I	1,450 (36.9)
II	897 (22.8)
III	1,532 (39.0)
IV	47 (1.2)
Mode of surgery, number (%)	
Open	1,493 (38.0)
Laparoscopy	2,433 (62.0)
Radicality of surgery, number (%)	
Curative	3,829 (97.5)
Palliative	97 (2.5)
Type of resection, number (%)	
Distal gastrectomy	1,967 (50.1)
Total gastrectomy	1,818 (46.3)
Proximal gastrectomy	141 (3.6)
Type of reconstruction, number (%)	
Billroth I	118 (3.0)
Billroth II	968 (24.7)
Roux-en-Y	2,299 (58.6)
Others	541 (13.8)
Duration of operation, min, number (%)	
<180	2,095 (53.4)
≥180	1,831 (46.6)

Data are presented as median (IQR) or frequency (percent).

ASA class, American Society of Anesthesiologists class; IQR, interquartile range.

**Table 2:** Anastomotic leakage site.

Anastomotic leakage site	Number (%)
Esophagojejunostomy	59 (73.8)
Esophagogastrostomy	9 (11.3)
Gastrojejunostomy	4 (5.0)
Gastroduodenostomy	3 (3.7)
Duodenal stump	5 (6.2)
Total	80 (100)

Data are presented as frequency (percent).

### 3.2 Diagnosis of anastomotic leakage

**Table 3** shows the approaches of AL diagnosis. 8.75% AL were diagnosed only by clinical presentation and biochemical analysis. Most AL was further confirmed *via* taking methylene blue orally (17.5%, 14/80), CT scan using intravenous contrast (83.75%, 67/80), or fluoroscopy using oral contrast (3.75%, 3/80). Moreover, the median time between surgery and AL diagnosis was 6 days (IQR 5–8 days).

**Table 3:** Diagnosis of anastomotic leakage.

Diagnostic method of leakage, number (%)	
Clinical presentation and biochemical analysis	7 (8.75)
CT scan	67 (83.75)
Fluoroscopy using oral contrast	3 (3.75)
Methylene blue	14 (17.5)
Time between surgery and leakage diagnosis (days), median (IQR)	6 (5-8)

Data are presented as median (IQR) or frequency (percent).

IQR, interquartile range.

### 3.3 Risk factors related to anastomotic leakage

Univariate analyses of the variables that were potentially associated with AL are demonstrated in **Table 4**. The presence of AL was remarkably universal among older patients (median 66 [IQR 60–70] vs. 63 [IQR 55–69 years], *P* = 0.008) and patients with diabetes (3.7 vs. 1.9%; *P* = 0.033). The lower the concentration of plasma albumin (median 37.1 [IQR 32.9–39.7] vs. 39.0 [IQR 36.3–41.7] g/l, *P* < 0.001), the higher the rate of AL, and similar results were observed in cholesterol (median 4.20 [IQR 3.53–4.73] vs. 4.47 [IQR 3.82–5.11] mmol/L, *P* = 0.013) and hemoglobin concentration (median 121.0 [IQR 107.5–137.8] vs. 130.0 [IQR 114.0–143.0] g/l, *P* = 0.025). Patients with history of previous laparotomy tended to develop AL more likely, although there was no significant difference (3.1 vs. 1.9%, *P* = 0.092). Moreover, the incidences of AL were significantly different depending on the tumor location (upper third: 4.4%, middle third: 1.7%, lower third: 0.7%, others: 0.5%, *P* < 0.001). The surgical factors influenced the AL development in many respects. Firstly, AL occurrence was remarkably more often in patients undergoing laparoscopic operation (open: 1.1%, laparoscopic: 2.6%, *P* = 0.002). Secondly, AL after total gastrectomy and proximal gastrectomy occurred more frequently than after distal gastrectomy (distal: 0.6%, total: 3.2%, proximal: 6.4%, *P* < 0.001). In addition, the rates of AL occurrence were significantly different in terms of type of reconstruction (Billroth I: 1.7%, Billroth II: 0.7%, Roux-en-Y: 2.7%, others: 1.8%, *P* = 0.005). Finally, when the duration of gastrectomy was more than or equal to 180 min, the AL incidence was significantly higher (≥180 min: 2.8%, <180 min: 1.3%, *P* = 0.001). Independent hazard factors related to AL were further revealed *via* multivariate analysis, including albumin concentration, diabetes, mode of surgery, and type of resection. The odds ratios, 95% confidence intervals, and *P*-values for these factors that had significant difference are presented in **Table 5**.

**Table 4:** Univariate analysis to identify clinicopathological and operative variables that are associated with anastomotic leakage.

	Leakage (n = 80)	No leakage (n = 3,846)	*P*
Age, years, median (IQR)	66 (60-70)	63 (55-69)	0.008*
Sex, number (%)			0.255
Male	63 (2.2)	2,782 (97.8)	
Female	17 (1.6)	1,064 (98.4)	
Body mass index, kg/m^2^, number (%)	24.0 (21.6-26.2)	23.3 (21.3-25.4)	0.547
ASA score, number (%)			1.000
<3	68 (2.0)	3,267 (98.0)	
≥3	12 (2.0)	579 (98.0)	
Hemoglobin concentration, g/L, median (IQR)	121.0 (107.5-137.8)	130.0 (114.0-143.0)	0.025*
Albumin concentration, g/L, median (IQR)	37.1 (32.9-39.7)	39.0 (36.3-41.7)	<0.001*
Total cholesterol, mmol/L, median (IQR)	4.20 (3.53-4.73)	4.47 (3.82-5.11)	0.013*
Lymphocyte count, ×10^9^/L, median (IQR)	1.59 (1.19-1.93)	1.5 (1.20-1.84)	0.900
Neutrophil count, ×10^9^/L, median (IQR)	3.35 (2.30-4.67)	3.39 (2.71-4.33)	0.395
Diabetes, number (%)			0.033*
Absent	66 (1.9)	3,477 (98.1)	
Present	14 (3.7)	369 (96.3)	
Smoking habits, number (%)			0.530
Non-smoker	55 (1.9)	2,772 (98.1)	
Smoker	25 (2.3)	1,074 (97.7)	
Drinking habits, number (%)			0.653
Non-drinker	65 (2.0)	3,192 (98.0)	
Drinker	15 (2.2)	654 (97.8)	
Previous laparotomy, number (%)			0.092
Absent	64 (1.9)	3,344 (98.1)	
Present	16 (3.1)	502 (96.9)	
Neoadjuvant chemotherapy, number (%)			0.183
Absent	79 (2.1)	3,663 (97.9)	
Present	1 (0.5)	183 (99.5)	
Tumor location, number (%)			<0.001*
Upper third stomach	50 (4.4)	1,096 (95.6)	
Middle third stomach	19 (1.7)	1,108 (98.3)	
Lower third stomach	10 (0.7)	1,446 (99.3)	
Others	1 (0.5)	196 (99.5)	
T stage, number (%)			0.474
T1	28 (2.1)	1,281 (97.9)	
T2	14 (2.7)	501 (97.3)	
T3	20 (1.6)	1,232 (98.4)	
T4	18 (2.1)	832 (97.9)	
N stage, number (%)			0.602
N0	32 (1.7)	1,827 (98.3)	
N1	12 (2.2)	540 (97.8)	
N2	13 (2.3)	544 (97.7)	
N3	23 (2.4)	935 (97.6)	
Metastasis, number (%)			1.000
M0	79 (2.0)	3,796 (98.0)	
M1	1 (2.0)	50 (98.0)	
Stage, number (%)			0.376
I	37 (2.6)	1,413 (97.4)	
II	16 (1.8)	881 (98.2)	
III	26 (1.7)	1,506 (98.3)	
IV	1 (2.1)	46 (97.9)	
Mode of surgery, number (%)			0.002
Open	17 (1.1)	1,476 (98.9)	
Laparoscopy	63 (2.6)	2,370 (97.4)	
Radicality of surgery, number (%)			0.449
Curative	77 (2.0)	3,752 (98.0)	
Palliative	3 (3.1)	94 (96.9)	
Type of resection, number (%)			<0.001*
Distal gastrectomy	12 (0.6)	1,955 (99.4)	
Total gastrectomy	59 (3.2)	1,759 (96.8)	
Proximal gastrectomy	9 (6.4)	132 (93.6)	
Type of reconstruction, number (%)			0.005*
Billroth I	2 (1.7)	116 (98.3)	
Billroth II	7 (0.7)	961 (99.3)	
Roux-en-Y	61 (2.7)	2,238 (97.3)	
Others	10 (1.8)	531 (98.2)	
Duration of operation, min, number (%)			0.001*
<180	28 (1.3)	2,067 (98.7)	
≥180	52 (2.8)	1,779 (97.2)	

Data are presented as median (IQR) or frequency (percent).

ASA class, American Society of Anesthesiologists class; IQR, interquartile range.

* indicates P<0.05.

**Table 5:** Multivariate analysis to identify clinicopathological and operative variables that are associated with anastomotic leakage.

	Multivariate analysis	
	Hazard ratio (OR, 95% *CI*)	*P*
Albumin concentration	0.917 (0.873-0.964)	0.001
Diabetes		
Absent	1	
Present	2.009 (1.094-3.690)	0.025
Mode of surgery		
Open	1	
Laparoscopy	3.172 (1.743-5.771)	<0.001
Type of resection		
Distal gastrectomy	1	
Total gastrectomy	3.593 (1.546-8.352)	0.003
Proximal gastrectomy	5.576 (1.856-16.750)	0.002

OR, odds ratio; CI, confidence interval.

### 3.4 Outcomes of conservative treatment

**Table 6** shows the results of conservative treatment. Of these 80 patients with AL, one (1.25%, 1/80) patient underwent emergency operation due to hemorrhage of the spleen on the 8th day after gastrectomy and two patients (2.5%, 2/80) received endoscopic intervention 1 and 3 months after gastrectomy, respectively. In addition, The median time from AL diagnosis to closure of AL was 17 days (IQR 11–26 days), the median time from AL diagnosis to discharge was 23 days (IQR 17–32 days), and the closure rate for conservative treatment of AL in the first month after AL diagnosis was 83.5% (66/79). 65% (52/80) and 32.5% (26/80) of patients with AL were diagnosed with hydrothorax and ascites, respectively. Thereinto, the proportions of patients undergoing thoracentesis and abdominocentesis were 38.8% (31/80) and 15% (12/80), respectively. According to the median time from AL diagnosis to closure, 77 patients only undergoing conservative treatment were divided into early recovery group (within 17 days, 36 patients) and late recovery group (17 or more days, 41 patients). As shown in **Table 7**, Univariate analysis demonstrated that the preoperative albumin concentration was remarkably higher in the early recovery group than in the late recovery group (*P* = 0.004).

**Table 6:** Outcome of conservative therapy for anastomotic leakage.

Management strategies of AL n (%)	
Conservative therapy	77 (96.3%, 77/80)
Surgery	1 (1.2%, 1/80)
Endoscopic invention	2 (2.5%, 2/80)
Time from leakage diagnosis to complete closure of leakage (days), median (IQR)	17 (11-26)
Time from leakage diagnosis to discharge (days), median (IQR)	23 (17-32)
Closure of leakage in 1 month after diagnosis	66 (83.5%, 66/79)
Complications n (%)	
Hydrothorax	52 (65.0%, 52/80)
Ascites	26 (32.5%, 26/80)
CT-guided percutaneous catheter drainage n (%)	
Thoracentesis	31 (38.8%, 31/80)
Abdominocentesis	12 (15.0%, 12/80)

Data are presented as median (IQR) or frequency (percent).

IQR, interquartile range.

**Table 7:** Univariate analyses to identify clinicopathological and operative variables that are associated with the time of conservative treatment.

	Time of conservative treatment <17 days (n = 36)	Time of conservative treatment ≥17 days (n = 41)	Univariate analyses
			*P*
Age, years, median (IQR)	64.5 (56.5-69)	67 (61-72)	0.156
Sex, number (%)			1.000
Male	29 (46.8)	33 (53.2)	
Female	7 (46.7)	8 (53.3)	
Body mass index, kg/m^2^, number (%)	25.5 (21.7-27.3)	23.5 (21.6-25.6)	0.214
ASA score, number (%)			0.528
<3	32 (48.5)	34 (51.5)	
≥3	4 (36.4)	7 (63.6)	
Hemoglobin concentration, g/L, median (IQR)	118.5 (100.5-142.5)	123.0 (115.0-137.0)	0.743
Albumin concentration, g/L, median (IQR)	39.2 (35.6-42.4)	36.4 (32.0-38.5)	0.004*
Total cholesterol, mmol/L, median (IQR)	4.18 (3.46-4.72)	4.19 (3.68-4.81)	0.797
Lymphocyte count, ×10^9^/L, median (IQR)	1.76 (1.43-1.93)	1.43 (1.11-1.93)	0.150
Neutrophil count, ×10^9^/L, median (IQR)	3.52 (2.35-4.99)	2.96 (2.37-4.25)	0.398
Diabetes, number (%)			0.557
Absent	31 (48.4)	33 (51.6)	
Present	5 (38.5)	8 (61.5)	
Smoking habits, number (%)			0.329
Non-smoker	27 (50.9)	26 (49.1)	
Smoker	9 (37.5)	15 (62.5)	
Drinking habits, number (%)			0.393
Non-drinker	31 (49.2)	32 (50.8)	
Drinker	5 (35.7)	9 (64.3)	
Previously laparotomy, number (%)			1.000
Absent	29 (46.8)	33 (53.2)	
Present	7 (46.7)	8 (53.3)	
Neoadjuvant chemotherapy, number (%)			1.000
Absent	36 (47.4)	40 (52.6)	
Present	0 (0.0)	1 (100.0)	
Tumor location, number (%)			0.167
Upper third stomach	22 (46.8)	25 (53.2)	
Middle third stomach	6 (31.6)	13 (68.4)	
Lower third stomach	7 (70.0)	3 (30.0)	
Others	1 (100.0)	0 (0.0)	
T stage, number (%)			0.602
T1	12 (44.4)	15 (55.6)	
T2	7 (50.0)	7 (50.0)	
T3	7 (36.8)	12 (63.2)	
T4	10 (58.8)	7 (41.2)	
N stage, number (%)			0.201
N0	11 (35.5)	20 (64.5)	
N1	6 (50.0)	6 (50.0)	
N2	8 (72.7)	3 (27.3)	
N3	11 (47.8)	12 (52.2)	
Metastasis, number (%)			0.468
M0	35 (46.1)	41 (53.9)	
M1	1 (100.0)	0 (0.0)	
Stage, number (%)			0.745
I	16 (44.4)	20 (55.6)	
II	7 (46.7)	8 (53.3)	
III	12 (48.0)	13 (52.0)	
IV	1 (100.0)	0 (0.0)	
Mode of surgery, number (%)			1.000
Open	7 (43.7)	9 (56.3)	
Laparoscopy	29 (47.5)	32 (52.5)	
Radicality of surgery, number (%)			0.098
Curative	33 (44.6)	41 (55.4)	
Palliative	3 (100.0)	0 (0.0)	
Type of resection, number (%)			0.246
Distal gastrectomy	7 (58.3)	5 (41.7)	
Total gastrectomy	23 (41.1)	33 (58.9)	
Proximal gastrectomy	6 (66.7)	3 (33.3)	
Type of reconstruction, number (%)			0.128
Billroth I	2 (100.0)	0 (0.0)	
Billroth II	2 (28.6)	5 (71.4)	
Roux-en-Y	25 (43.1)	33 (56.9)	
Others	7 (70.0)	3 (30.0)	
Duration of operation, min, number (%)			1.000
<180	13 (48.1)	14 (51.9)	
≥180	23 (46.0)	27 (54.0)	
Diagnostic method of leakage			0.99
CT scan	17 (45.9)	20 (54.1)	
Clinical presentation and biochemical analysis	11 (47.8)	12 (52.2)	
Fluoroscopy using oral contrast	1 (33.3)	2 (66.7)	
Methylene blue	3 (50.0)	3 (50.0)	
CT scan and methylene blue	4 (50.0)	4 (50.0)	
Anastomotic leakage site			0.23
Esophagojejunostomy	23 (41.1)	33 (58.9)	
Esophagogastrostomy	6 (66.7)	3 (33.3)	
Gastrojejunostomy	1 (25.0)	3 (75.0)	
Gastroduodenostomy	2 (66.7)	1 (33.3)	
Duodenal stump	4 (80.0)	1 (20.0)	

Data are presented as median (IQR) or frequency (percent).

ASA class, American Society of Anesthesiologists class; IQR, interquartile range. * indicates P<0.05.

### 3.5 Relation between anastomotic leakage and overall survival

**Figure 1:**
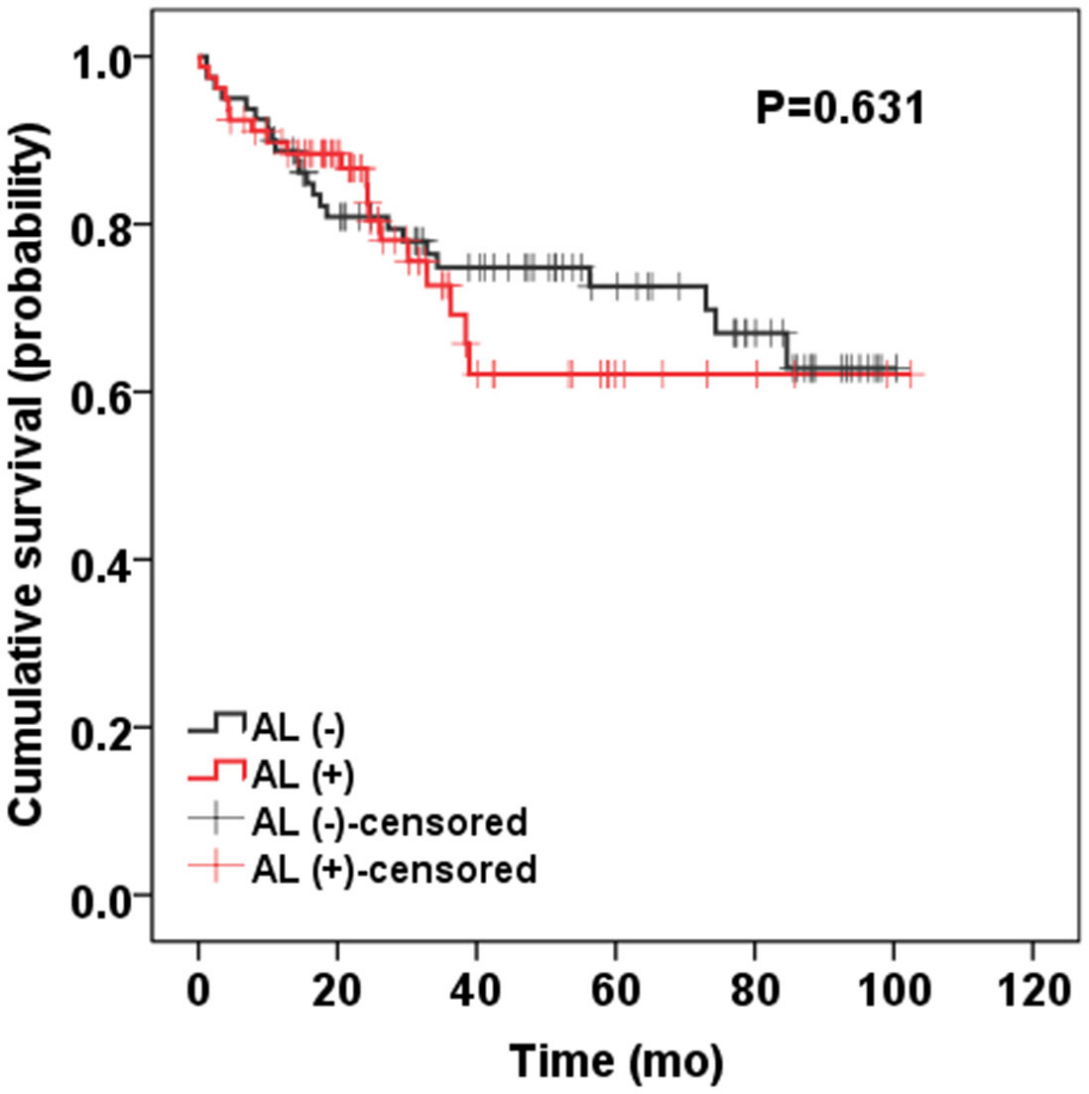
Kaplan–Meier curve presenting the overall survival rate.

In order to evaluate the effect of AL on overall survival, the propensity score matching (PSM) method was utilized to balance the baseline features of two groups of patients with or without AL. Propensity scores were calculated according to age, sex, preoperative treatment, TNM stage, and extent of resection, with a ratio of 1:1. Totally, 11 patients (13.75%, 11/80) with AL died, compared with 20 patients (25%, 20/80) without AL (*P* = 0.109). **Figure 1** shows that the overall mean survival had no significant difference between patients with and without AL: 75.78 (95% CI 67.28–84.23) vs. 72.43 (95% CI 61.00–82.36) months, *P* = 0.631.

## 4 Discussion

The present study indicated that the AL rate of gastric cancer patients after gastrectomy was relatively low (2.03%), similar to previously reported studies (1.0%~4.2%) (12–14, 23–25). In addition, one patient died of septic shock caused by intraperitoneal abscess and pulmonary infection 4 months after gastrectomy, and the incidence of AL-related death was only 1.25% (1/80). Several large cohort studies demonstrated that mortality associated with AL ranged from 0% to 7.0% (15, 16, 26).

Exploration of risks for AL is conducive to the treatment and prevention of AL. At present, comorbidities, neoadjuvant treatment, anastomotic location, surgical and anastomotic technique, perioperative monitoring, and therapy are considered as the most possible risk factors for the occurrence of AL (27–31). Due to lack of general agreement on the most important risk factors, there were reliable predictive tools for risk evaluation of AL occurrence. Kim et al. (16) demonstrated that gender, heart disease, location of tumor, and transfusion might promote the incidence of AL. Roh et al. (15) reported that lack of experience might have an adverse effect on AL development. In the present study, univariate analyses indicated that in the elderly, the low concentrations of plasma hemoglobin, albumin, and cholesterol, diabetes, tumors located in the upper third stomach, the laparoscopic approach, proximal or total gastrectomy, esophagojejunostomy, and long operation time were supposed to promote the presence of AL. Multivariate analysis revealed that albumin concentration, diabetes, the laparoscopic approach, and proximal or total gastrectomy were the independent risk factors facilitating AL development. Esophagojejunostomy, accounting for 73.8% of AL, was the most frequent site of AL, which reflected that tumor location in the lower third stomach and distal gastrectomy with Billroth I or II anastomosis were the low risk factors for AL occurrence. In light of the complexity and difficulty of esophagojejunostomy, previous studies have proposed various techniques to prevent AL development (32–34). Laparoscopic gastrectomy has been widely conducted to treat gastric cancer (35, 36); however, minimally invasive surgery demands adequate practice and sufficient experience to avoid risks of AL (37). Therefore, patients should be selected to undergo laparoscopic gastrectomy according to body weight, tumor stage, and comorbidities. In a short period, our surgical team performed totally laparoscopic gastrectomy on patients with advance gastric cancer of esophagogastric junction, and this may result in the fact that the laparoscopic approach contributed to AL development.

Owing to lack of specific clinical signs, it is difficult to diagnose AL after gastrectomy, and the sensitive and specific diagnostic modality of AL remains unavailable (38–40). Except for clinical presentation, biochemical analysis of the levels of blood inflammation biomarkers, such as white blood cell counts, C-reactive protein, and procalcitonin, is a useful predictor of AL (41). Moreover, drain amylase levels can reflect the possibility of AL (42). In the present study, CT scan with intravenous contrast is utilized to screen most of the patients. In addition to helping to diagnose AL, CT scan with contrast can supply useful information in terms of peri-anastomotic fluid accumulation. In addition, this method can help confirm sepsis resulting from other types of intra-abdominal complications and contribute to decide the necessity of percutaneous drainage (43, 44). Along with the development of endoscopic techniques, endoscopy has become a reliable diagnostic tool with almost 95% specificity and sensitivity (40). Intraoperative endoscopic examination can directly detect AL in the esophagojejunostomy site, but the accuracy is affected by the proficiency of the endoscopist. It is important to diagnose AL timely and effectively for the reason that the incidence of fatal complications, the length of hospitalization, and the medical cost can be greatly reduced. The study by Kim et al. (26) indicated that the median time between surgery and diagnosis of AL was 8 days (IQR, 6–13 days). At present, the outcome was 6 days (IQR 5–8 days).

Treatments of AL were classified as conservative strategies, surgical methods, and endoscopic management (45). Because of complex and changeable clinical manifestation, the standardized treatment strategies remain unclear. The key points of AL management are the closure or contraction of the anastomotic defect to prevent peritoneal contamination and reduce fluid accumulation. However, AL size and location, symptom degree, the occurrence of necrosis, and time between surgery and diagnosis of AL all have an influence on the choice of treatment strategies (45). Despite lack of a consensus guideline, the initial treatment has a shift from operation to conservative strategies and endoscopic therapy (11, 20). Along with the development of techniques, endoscopic treatment has gradually been applied more and more widely. Numerous studies had proved the efficacy of endoscopic intervention (19, 21). The study by Kim et al. (26) reported that the rate of complete AL closure by using endoscopic treatment was 80% and the failure of AL closures was related to location of AL and the occurrence of intra-abdominal abscess. Nevertheless, the superiority of endoscopic treatment compared with conservative strategies is still unclear. In this study, most patients (96.3%, 77/80) underwent conservative treatment. The median time from AL diagnosis to closure of AL was 17 days (IQR 11–26 days), the median time from AL diagnosis to discharge was 23 days (IQR 17–32 days), and the closure rate for conservative treatment of AL in the first month after AL diagnosis was 83.54% (66/79). These outcomes were equal to or better than results of previous studies.

The present study still has some limitations. Firstly, there are no uniform criteria on the treatment success. Some studies assessed the therapeutic efficacy *via* endoscopy, and endoscopic examinations were conducted every 3 or 7 days (15, 26). The success of AL treatment was defined as the complete AL closure. In our study, when the clinical signs turned up, for instance, fever or peritonitis disappeared, the level of blood inflammatory biomarkers returned to normal, the abdominal abscess and hydrothorax shrunk or even disappeared, or there was no abnormal drainage, the closure of AL was considered. Another limitation of our study was that the AL rate is lower than that of studies in the west (46), and this difference might result from patients’ characteristics including age, comorbidity, and tumor stage. 

## 5 Conclusion

In conclusion, AL after gastrectomy is associated with diabetes, operation method, extent of resection, and duration of operation. Conservative treatment is relatively safe and effective for the management of AL in patients after gastric cancer surgery.

## Data availability statement

The original contributions presented in the study are included in the article/supplementary material. Further inquiries can be directed to the corresponding author.

## Ethics statement

The studies involving human participants were reviewed and approved by the First Affiliated Hospital of Nanjing Medical University. Written informed consent for participation was not required for this study in accordance with the national legislation and the institutional requirements.

## Author contributions

ZH and ZX designed the study. QL and HL performed the data extraction. HL and LZ conducted the analysis. ZH wrote the original manuscript. HX, DZ, and LW critically revised the manuscript. All authors contributed to the study approved the submitted version.
